# Supplementary material for: Short-Term Outcome of Robotic versus Laparoscopic Hysterectomy for Endometrial Cancer in Women with Diabetes: Analysis of the US Nationwide Inpatient Sample
Source: J Clin Med. 2023 Dec 15;12(24):7713. doi: 10.3390/jcm12247713 (PMC10743688; doi:10.3390/jcm12247713)
Supplement: Supplementary file 1 [file jcm-12-07713-s001.zip › jcm-2708788-supplementary.pdf]

Supplementary Table S1. ICD codes used in the analyses.

	ICD-9-CM / ICD-9-PCS	ICD-10-CM / ICD-10-PCS
Endometrial cancer	182.x	C54.x
Diabetes	250	E10, E11
Laparoscopic	ICD9-PCS: 68.31, 68.41, 68.5, 68.61, 68.7	ICD10-PCS: 0UT94ZL, 0UT94ZZ, 0UT97ZL, 0UT97ZZ, 0UT98ZL, 0UT98ZZ, 0UT9FZL, 0UT9FZZ, 0UT44ZZ, 0UT47ZZ, 0UT48ZZ
Robotic	ICD9-PCS: Any of 68.3x, 68.4x, 68.5x, 68.6x, 68.7x, 68.9x, and with17.4	ICD10-PCS: Any of 0UT94ZL, 0UT94ZZ, 0UT97ZL, 0UT97ZZ, 0UT98ZL, 0UT98ZZ, 0UT9FZL, 0UT9FZZ,0UT44ZZ, 0UT47ZZ, 0UT48ZZ, and with any of 8E0W0C*, 8E0W3C*, 8E0W4C*, 8E0W7C*, 8E0W8C*
Lymph node invasion or metastatic disease	196-199	C77-C79
AMI	410	I21
CVA	433.01, 433.10, 433.11, 433.21, 433.31, 433.81, 433.91, 434.00, 434.01, 434.11, 434.91, 436, 430, 431	I60, I61, I63, I69
VTE	415, 451-453, 671, 673, 997.2	I260, I269, I801-803, I808, I809, I820-I823, I828, I829, O082, O223, O871, O882, I81, I82
Pneumonia	486, 481, 482.8, 482.3, 484, 115.05, 115.15, 115.95	A48.1, J12 - J18, B39.2, B39.5, B39.9, A22.1, B25.0, A37.91, B44.0
Sepsis	995.9, 996.64, 038, 999.3, 790.7, 041.x, 785.52	R78.81, A41, R65.2, T81.4, T80.2, A42.7, A22.7, B37.7, A26.7, A28.2, A54.86, B00.7, A32.7, A24.1, A39.2, A20.7, A21.7, A48.3
Surgical site infection	998.5	T81.4
Major blood loss	459.0, 285.1, 998.1<br>ICD9-PCS: 39.98, 99.0	R58, D62<br>ICD10-PCS: 0W380ZZ, 0W383ZZ, 0W384ZZ, 302*

Respiratory failure/ mechanical ventilation	518.5, 518.81-518.84<br>ICD9-PCS: 96.7, 93.90, 96.01-96.05	J95.2-J95.8, J96.00, J96.90, J80, J81.0<br>ICD10-PCS: 5A1935Z, 5A1945Z, 5A1955Z, 5A09357, 5A09457, 5A09557, 09HN7BZ, 09HN8BZ, 0CHY7BZ 0CHY8BZ, 0DH57BZ, 0DH58BZ 0BH17EZ, 0BH18EZ, 0B717DZ 0B718DZ, 0BH07DZ
Wound dehiscence	998.3, 998.83, 998.89, 998.9	T81.3, T81.89, T81.9
AKI	584.x	N17
UTI	593.3	N39.0
Smoking	305.1, V15.82, 989.84	Z71.6, Z72.0, Z86.43, Z87.891, F17, O99.33, T65.2
Obesity	278.00, 278.01, V85.3, V85.4	E66.01, E66.09, E66.1, E66.2, E66.8, E66.9, Z68.3, Z68.4
CKD	403.01, 403.11, 403.91, 404.02, 404.03, 404.12, 404.13, 404.92, 404.93, 582, 583.0-583.7, 585, 586, 588.0, V42.0, V45.1, V56	I12.0, I13.1, N03.2-N03.7, N05.2-N05.7, N18, N19, N25.0, Z49.0-Z49.2, Z94.0, Z99.2
Ischemic heart disease	410–414	I25
Congestive heart failure	CM_CHF=1	I09.9, I11.0, I13.0, I13.2, I25.5, I42.0, I42.5-I42.9, I43, I50, P29.0
Atrial fibrillation	427.31	I48
Anemia	CM_ANEMDEF=1	D60, D61, D63, D64
COPD	491, 492, 496<br>CM_CHRNLUNG=1	J40-44
Cerebrovascular disease	362.34, 430.x-438.x	G45, G46, H34.0, I60-I69
Peripheral vascular disease	093.0, 437.3, 440.x, 441.x, 443.1-443.9, 447.1, 557.1, 557.9, V43.4	I70.x, I71.x, I73.1, I73.8, I73.9, I77.1, I79.0, I79.2, K55.1, K55.8, K55.9, Z95.8, Z95.9

Severe Liver disease	456.0–456.2, 572.2–572.8;	I85.0, I85.9, I86.4, I98.2, K70.4, K71.1, K72.1, K72.9, K76.5, K76.6, K76.7
Rheumatic disease	446.5, 710.0-710.4, 714.0-714.2, 714.8, 725<br>CM_ARTH=1	M05, M06, M31.5, M32-M34, M35.1, M35.3, M36.0
Coagulopathy	CM_COAG=1	D65-69
Diabetes with chronic complication	250.4-250.7	E10.2-E10.5, E10.7, E11.2-E11.5, E11.7, E12.2-E12.5, E12.7, E13.2-E13.5, E13.7, E14.2-E14.5, E14.7

AMI—acute myocardial infarction; CVA—cerebrovascular accident; VTE—venous thromboembolism; AKI—acute kidney injury; UTI—urinary tract infection; CKD—chronic kidney disease; COPD—chronic obstruction pulmonary disease; ICD—International Classification of Diseases; CM—clinical modification; PCS—procedure coding system.

Supplementary Table S2. Full model of the associations between study variables and in-hospital outcomes in women with diabetes undergoing minimally invasive hysterectomy for endometrial cancer.

	Any complication	Unfavorable discharge [a]	LOS [a]	Hospital cost, US dollars
**Variables**	aOR (95% CI)	aOR (95% CI)	aBeta (95% CI)	aBeta (95% CI)
Robotic (vs pure laparoscopic)	0.88 (0.75, 1.04)	**0.63 (0.46, 0.85)**	**-0.46 (-0.57, -0.35)**	**6129.93 (4448.74, 7811.12)**
Age, years (vs. 18-49)				
50-59	1.14 (0.84, 1.55)	1.62 (0.68, 3.88)	-0.45 (-1.14, 0.25)	-2707.76 (-5548.35, 132.03)
60-69	1.10 (0.82, 1.47)	1.60 (0.71, 3.88)	-0.42 (-1.13, 0.29)	-2234.68 (-5215.94, 746.59)
70-79	1.29 (0.94, 1.77)	**1.60 (0.71, 3.61)**	-0.27 (-1.03, 0.29)	-668.07 (-3905.24, 2569.10)
≧80	**2.11 (1.48, 3.00)**	**2.90 (1.28, 6.58)**	0.19 (-0.55, 0.93)	596.75 (-2320.24, 3513.74)
Race (vs. White)				
Black	1.16 (0.93, 1.45)	0.91 (0.60, 1.38)	**0.56 (0.43, 0.69)**	**3161.09 (1907.90, 4414.29)**
Hispanic	0.99 (0.78, 1.26)	0.66 (0.38, 1.15)	**0.46 (0.003, 0.91)**	**8115.64 (6081.98, 10149.30)**
Other	0.92 (0.67, 1.26)	0.68 (0.35, 1.34)	0.05 (-0.02, 0.13)	**4119.55 (2981.20, 5257.89)**
Insurance status (vs. Medicare/Medicaid)				
Private including HMO	**0.69 (0.57, 0.83)**	**0.20 (0.11, 0.36)**	**-0.47 (-0.67, -0.27)**	**-2558.70 (-3981.70, -1135.70)**

Self-pay/no-charge/other	0.93 (0.66, 1.32)	**0.10 (0.01, 0.71)**	-0.28 (-0.60, 0.05)	**-8168.11 (-9758.39, -6577.83)**
Household income (vs. Q1)				
Q2	1.03 (0.85, 1.25)	1.34 (0.92, 1.94)	0.05 (-0.14, 0.24)	-558.01 (-1533.67, 417.66)
Q3	0.82 (0.67, 1.02)	1.20 (0.80, 1.80)	-0.13 (-0.35, 0.08)	**-4581.88 (-5995.60, -3168.15)**
Q4	0.82 (0.66, 1.02)	1.32 (0.88, 1.97)	-0.09 (-0.31, 0.13)	**-1836.03 (-3184.03, -488.03)**
Smoking (yes vs. no)	1.09 (0.91, 1.30)	1.00 (0.72, 1.38)	0.02 (-0.04, 0.09)	**-878.39 (-1614.67, -142.11)**
Obesity (yes vs. no)	**1.55 (1.34, 1.80)**	**2.00 (1.53, 2.61)**	**0.53 (0.36, 0.70)**	**6745.39 (5664.38, 7826.41)**
Study year (vs. 2005-2009)				
2010-2015	**1.58 (1.22, 2.04)**	1.26 (0.81, 1.94)	-0.02 (-0.40, 0.35)	**12107.75 (9309.21, 14906.30)**
2016-2018	1.31 (0.98, 1.75)	1.12 (0.68, 1.85)	-0.35 (-0.72, 0.02)	**25551.55 (23134.71, 27968.39)**
Hospital region (vs. Northeast)				
South	1.02 (0.80, 1.29)	0.95 (0.65, 1.37)	-0.19 (-0.41, 0.02)	1571.24 (-900.80, 4043.29)
Midwest	1.06 (0.85, 1.33)	**0.67 (0.46, 0.97)**	-0.28 (-0.59, 0.02)	**3170.80 (548.07, 5793.53)**
West	1.03 (0.80, 1.32)	0.77 (0.52, 1.15)	**-0.40 (-0.69, -0.11)**	**20822.10 (18298.15, 23346.04)**
Hospital location/teaching status (vs. Urban teaching)				
Rural	**0.47 (0.39, 0.57)**	0.88 (0.53, 1.44)	**-0.64 (-0.78, -0.50)**	**-20906.34 (-22381.85, -19430.82)**
Urban nonteaching	0.90 (0.74, 1.09)	0.89 (0.63, 1.26)	**0.10 (0.04, 0.16)**	**2578.56 (1869.84, 3287.28)**
CKD (yes vs. no)	**1.53 (1.12, 2.07)**	**2.14 (1.20, 3.81)**	**0.69 (0.34, 1.04)**	**11804.01 (9261.48, 14346.55)**
Peripheral vascular disease (yes vs. no)	**1.75 (1.17, 2.63)**	1.67 (0.97, 2.87)	**0.09 (0.003, 0.18)**	**5441.53 (3814.63, 7068.43)**
Diabetes with chronic complications (yes vs. no)	**2.22 (1.78, 2.75)**	**1.90 (1.23, 2.94)**	**1.10 (0.92, 1.28)**	**10077.73 (8482.62, 11672.83)**
Emergency admission (yes vs no)	**2.09 (1.70, 2.56)**	**1.75 (1.24, 2.47)**	**-1.68 (-2.21, -1.14)**	**-16414.45 (-20369.32, -12459.59)**

CKD—chronic kidney disease; aOR—adjusted odds ratio; ref—reference; LOS—length of stay; CI—confidence interval. Variables with p-values < 0.05 are shown in bold. a Excluded patients with in-hospital mortality.
